# Cosmetics as a Feature of the Extended Human Phenotype: Modulation of the Perception of Biologically Important Facial Signals

**DOI:** 10.1371/journal.pone.0025656

**Published:** 2011-10-03

**Authors:** Nancy L. Etcoff, Shannon Stock, Lauren E. Haley, Sarah A. Vickery, David M. House

**Affiliations:** 1 Department of Psychiatry, Massachusetts General Hospital, Boston, Massachusetts, United States of America; 2 Harvard Medical School, Cambridge, Massachusetts, United States of America; 3 Department of Biostatistics and Computational Biology, Dana-Farber Cancer Institute, Boston, Massachusetts, United States of America; 4 Procter & Gamble Beauty & Grooming, Procter & Gamble Cosmetics, Hunt Valley, Maryland, United States of America; 5 Department of Computer Sciences, Boston University, Boston, Massachusetts, United States of America; Nothwestern University, United States of America

## Abstract

Research on the perception of faces has focused on the size, shape, and configuration of inherited features or the *biological phenotype*, and largely ignored the effects of adornment, or the *extended phenotype*. Research on the evolution of signaling has shown that animals frequently alter visual features, including color cues, to attract, intimidate or protect themselves from conspecifics. Humans engage in conscious manipulation of visual signals using cultural tools in real time rather than genetic changes over evolutionary time. Here, we investigate one tool, the use of color cosmetics. In two studies, we asked viewers to rate the same female faces with or without color cosmetics, and we varied the style of makeup from minimal (natural), to moderate (professional), to dramatic (glamorous). Each look provided increasing luminance contrast between the facial features and surrounding skin. Faces were shown for 250 ms or for unlimited inspection time, and subjects rated them for attractiveness, competence, likeability and trustworthiness. At 250 ms, cosmetics had significant positive effects on all outcomes. Length of inspection time did not change the effect for competence or attractiveness. However, with longer inspection time, the effect of cosmetics on likability and trust varied by specific makeup looks, indicating that cosmetics could impact automatic and deliberative judgments differently. The results suggest that cosmetics can create supernormal facial stimuli, and that one way they may do so is by exaggerating cues to sexual dimorphism. Our results provide evidence that judgments of facial trustworthiness and attractiveness are at least partially separable, that beauty has a significant positive effect on judgment of competence, a universal dimension of social cognition, but has a more nuanced effect on the other universal dimension of social warmth, and that the extended phenotype significantly influences perception of biologically important signals at first glance and at longer inspection.

## Introduction

First impressions based on facial appearance occur automatically, are difficult to overcome, and impact decision-making. The strong motivational influence of facial beauty has been shown in studies of labor markets suggesting that there is a ”beauty premium” and “plainness penalty” [1] such that attractive individuals are more likely to be hired, promoted, and to earn higher salaries than unattractive individuals [2]–[4]. Social psychologists have identified a “halo” effect of beauty leading to a range of positive inferences including that the beautiful are more socially skilled, confident and successful [5]–[7]. Inferences of another attribute, competence, gleaned from a one second exposure to faces of unknown congressional candidates predict their electoral success [8], [9]. Players in a trust game invested more money in individuals whose faces were rated as trustworthy, despite the fact that there is no objective relationship between facial appearance and actual behavior [8].

Darwinian approaches posit that features of beautiful faces are important biological signals of mate value that motivate behavior in others, and have identified features such as averageness, symmetry and sexual dimorphism as key contributors to female facial beauty [5]–[7], [10], [11]. It is less clear what visual facial attributes lead to rapid judgments of trustworthiness, competence, and likeability from the face. Although the “beauty halo” may provide a partial explanation, it is likely that cues from facial expressions (real or mimicked by the contours of natural features), and facial immaturity or maturity are important drivers, the first signaling friendly or hostile intent (positive emotion or anger), and the latter, the perceived ability to carry out one's intentions [12]–[14]. All such facial judgments occur quickly, reliably, and change little with inspection time, suggesting that they are effortless and automatic. Recent models of social cognition and decision-making distinguish between such fast operating, reflexive processes or “system 1 processes” and slower, deliberate, effortful, and reflective "system 2" processes [15], [16].

To date, face research has focused largely on the *biological phenotype*, such as the shape, size, configuration and movement of facial features, and ignored the effects of facial adornment and grooming, or “the *extended phenotype*.” The extended phenotype [17], [18] refers to any effect of the genes beyond the organism's body. The spider's web, the hermit crab's shell, the bowerbird's bower and the beaver's dam are all considered examples of the extended phenotype. Among human artifacts, clothing, makeup and other forms of body adornment are considered phenotypic extensions. They are found universally and presumed to enhance perceived biological fitness.

Research on the evolution of signaling has shown that animals frequently alter or exaggerate visual features, including color cues, to attract, mimic, intimidate or protect themselves from conspecifics, sometimes setting off an arms race between deception and the detection of such deception [19]. Humans engage in conscious manipulation of visual signals using cultural tools in real time rather than genetic changes over evolutionary time. These tools highlight, exaggerate or conceal features of the heritable phenotype and are intended to modulate social impressions and confer advantages. But how effective they are, for what purposes, and by which mechanisms, are questions that have been minimally explored.

Experimental studies by Tinbergen first showed that it was possible to exaggerate a visual sign stimulus and produce a super-normal stimulus that elicits a super-normal response [20]. For example, herring gull chicks will peck more forcefully to red knitting needles than to a normal herring gull beak: the needles offer exaggerations of the shape and color cues found on the mother's beak. Supernormal stimuli have been found for many species. It may be that by isolating and exaggerating pre-existing cues to attractiveness and exploiting human sensory biases, adornments can heighten and exaggerate our normal aesthetic responses, rendering the adorned face or body a supernormal stimuli and our responses, supernormal responses.

Here we examine one example of the human extended phenotype, the use of color cosmetics, a tool used primarily by women to enhance facial attractiveness. The use of cosmetics is ancient. Analyses of Egyptian cosmetic powders dated from 1200 to 200 BC show that very sophisticated wet chemical technology was already being used in their creation [21]. The ancient Egyptians had versions of most of the cosmetics that we have today [22]. Interestingly, the use of cosmetics rose precipitously with the advent of photography [23], suggesting that both may serve as tools in an escalating beauty arms race. Today cosmetic use is ubiquitous. In a 2010 survey, the majority (63%) of women ages 18 or older in the United States reported that they had used some type of makeup product during the past year [24]. The global color cosmetics market is projected to reach $41.4 billion by the year 2015 [25]. Surprisingly few studies have investigated their effects on perceivers. The few that have demonstrate that cosmetics can increase attractiveness in Caucasian women in their 20s and 30s [26]–[29]. Research on inferences of personality and character has yielded conflicting and inconclusive findings [28]–[31].

As popular agents of self-advertising, cosmetics have been subject to shifting cultural attitudes toward their use. They were apparently considered so good at deceiving husbands In the late eighteenth century, and so feared by them, that the English government proposed a law stating that, “All women…that shall from and after this act impose upon, seduce or betray into matrimony any of his Majesty's subjects by the use of scents, paints, cosmetics, washes, … shall incur the penalty of the law now in force against witch craft and like misdemeanors and that the marriage upon convictions shall stand null and void” [22]. Over the centuries, women debated whether they were the “province of sophisticated beauties or the downfall of wanton souls.” [23], or tools used by admired and envied, or potentially untrustworthy individuals. Cosmetics have been subject to “display rules” with practices ranging from restrictive to permissive. Like fashion in clothing, norms for what makeup looks are considered most attractive change.

In current culture, cosmetics are seen as freely chosen and morally neutral agents of beauty enhancement. Their use reflects the individual's preferences and choices, and the response to their use reflects the perceiver's attitudes about forms of self-presentation and grooming practices. Thus, when viewing a face with makeup, perceivers make inferences based not only on cosmetics' effects on the appearance of symmetry, clearness of skin or featural contrast, but on their conscious ideas about makeup use and what it may signify about the user's personality, character, and intentions.

We hypothesize that cosmetics will impact face perception at a system 1 and system 2 levels, engaging both a reflexive and a reflective response. We predict that at an automatic, implicit level, cosmetics will have uniformly positive effects on judgments of beauty, personality and character. We predict that with longer inspection time, cosmetics will continue to enhance attractiveness, but may no longer uniformly enhance judgments of likability or trust, given different social attitudes toward cosmetics use. On longer inspection we expect to find greater beauty, but not necessarily the halo surrounding it for all looks.

Finally, to measure one potential source of the cosmetic effect on face perception we measured luminance contrast between the eyes, lips, and the surrounding skin for faces without makeup and for each of the makeup looks. Russell [26], [32] has found that sex differences in facial contrast influence the perception of facial gender: an androgynous face can be made to appear female by increasing the facial contrast, or to appear male by decreasing the facial contrast. Further, female faces wearing cosmetics had greater facial contrast than the same faces not wearing cosmetics, suggesting that cosmetics may function in part by exaggerating a sexually dimorphic attribute—facial contrast.

## Methods

### Ethics Statement

We have obtained ethics approval for our study from the Partners Human Research Committee at Massachusetts General Hospital, where participants were recruited and human experimentation was conducted. This project meets the criteria for exemption from further IRB review per the regulations found at 45 CFR 46.101(b) (2) Use of educational tests (cognitive, diagnostic, aptitude, achievement), survey procedures, interview procedures, or observation of public behavior. Instead of a consent form, we were instructed to provide subjects with a fact sheet and not collect a signature. The individuals/subjects pictured in this manuscript have given written informed consent to publication of their case details. By signing our consent form and photo release, they permit us to publish or make other public use of their facial digital images with the understanding that their names and/or personal information will not be made public.

To test the impact of cosmetics on judgments of faces we conducted two studies in which the same models were judged with and without makeup.

### Participants

Subjects in the first study included 149 adults (61 men, 88 women) of different ethnicities who were shown the faces for 250 ms. The second study included 119 adults (30 males, 89 females) of different ethnicities given unlimited time to inspect each face.

### Stimuli

**Figure 1:**
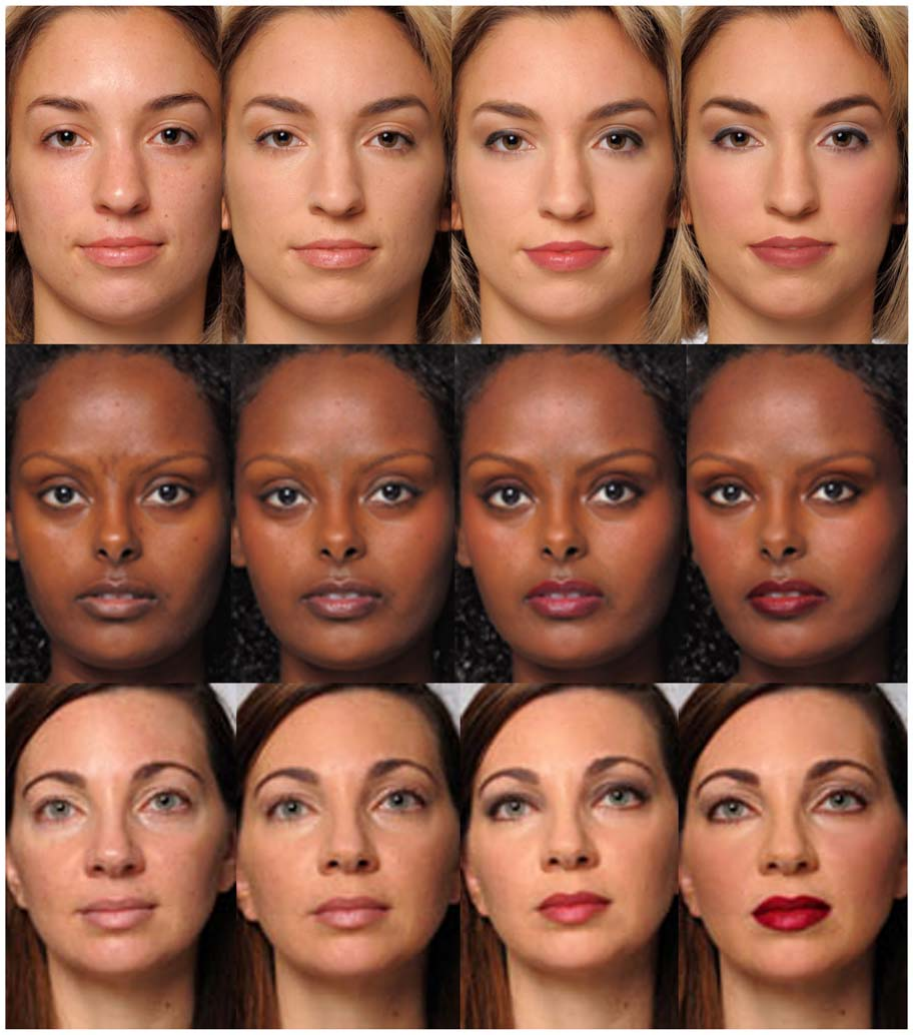
Models without makeup and with natural, professional and glamorous makeup.

The 100 high-resolution color images were of 25 women ages 20–50, self-identified as Hispanic, Caucasian, or African American shown in a frontal headshot with a neutral facial expression, and cropped to remove clothing and hairstyle. We used female models only, as they represent the vast majority of facial cosmetic users. A professional photographer took the images under uniform conditions. Makeup was applied by a makeup artist and then adjusted digitally. Each model was photographed without makeup and with 3 makeup looks ranging from minimal to moderate to dramatic, looks we informally labeled “natural”, “professional” or “glamorous” (Figure 1). We never used these labels during our work with models or with subjects. Finally, to eliminate demeanor cues that may result from the models seeing themselves with makeup, we removed all mirrors from the studio.

### Procedure

Subjects were presented the stimuli on an iMac desktop. Each subject saw all four looks for each of the 25 models in a randomized sequence and rated them for attractiveness, likeability, trustworthiness, and competence using a 7-point “slider scale” ranging from end points labeled “not at all” to “highly/extremely.”

## Results

### Analysis

Our statistical analyses of makeup by inspection time were obtained from a linear mixed effects model with crossed random effects, similar to the model described by Baayen et al. [33]. We conducted two analyses-the first aggregated the makeup looks and compared any use of cosmetics to no makeup. The second compared each individual makeup look to no makeup. We used a Bonferroni correction to preserve the overall type-1 error rate of 0.05 for the pair wise comparisons used within each analysis.

Overall, makeup (aggregated across all looks) produced a significant positive main effect on judgments of all outcomes in the 250 ms presentation (all p<0.0001) and on judgments of attractiveness, p<0.0001), competence (p<0.0001), and likeability (p<0.0001) , but not trustworthiness on longer inspection (p = 0.8988) (Table 1, Bonferroni corrected significance level  = 0.05/8 = 0.006). A significant interaction of makeup x inspection time revealed that perceptions of likability and trustworthiness showed a significantly larger positive makeup effect at 250 ms presentation times than when presented with unlimited inspection time: likeability (F(1,27000) = 10.71, p = 0.0011) and trustworthiness (F(1,27000) = 10.85, p = 0.0010) Length of inspection time did not modify the strength of the makeup effect for competence or attractiveness (Table 2).

**Table 1:** Comparisons of mean outcome scores for makeup (aggregated) versus no makeup obtained from the regression models (see Table 2).

Inspection Time	Outcome	Contrast	Estimate	SE	t-Statistic	DF	P
**250ms**	Competence	Makeup vs. No Makeup	0.34	0.02	15.98	26506	<0.0001
	Likability	Makeup vs. No Makeup	0.2	0.02	9.15	26506	<0.0001
	Attractiveness	Makeup vs. No Makeup	0.66	0.02	30.5	26506	<0.0001
	Trustworthiness	Makeup vs. No Makeup	0.11	0.02	5.08	26506	<0.0001
**Unlimited**	Competence	Makeup vs. No Makeup	0.3	0.02	12.61	26506	<0.0001
	Likability	Makeup vs. No Makeup	0.09	0.02	3.8	26506	0.0001
	Attractiveness	Makeup vs. No Makeup	0.64	0.02	26.22	26506	<0.0001
	Trustworthiness	Makeup vs. No Makeup	0	0.02	0.13	26506	0.8988

**Table 2:** Regression models aggregating the makeup looks.

Outcome	Covariate			Estimate	SE	t-Stat.	DF	P	F-Stat.	Num. DF; Den. DF	P
Competence	Intercept			3.87	0.12	33.53	65.95	<0.0001			
	ModelMakeup	Makeup		0.30	0.02	12.61	26506.06	<0.0001	402.04	1;27000	<.0001
		No Makeup		0.00							
	length		250 ms	-0.12	0.10	-1.24	300.10	0.2167	1.13	1;270	0.2889
			Unlimited	0.00							
	ModelMakeup*length	Makeup	250 ms	0.04	0.03	1.26	26506.04	0.2079	1.59	1;27000	0.2079
			Unlimited	0.00							
		No Makeup	250 ms	0.00							
			Unlimited	0.00							
Likability	Intercept			3.74	0.14	26.50	44.19	<0.0001			
	ModelMakeup	Makeup		0.09	0.02	3.80	26506.06	0.0001	79.83	1;27000	<.0001
		No Makeup		0.00							
	length		250 ms	-0.01	0.10	-0.14	304.32	0.8893	0.19	1;270	0.6673
			Unlimited	0.00							
	ModelMakeup*length	Makeup	250 ms	0.11	0.03	3.27	26506.03	0.0011	10.71	1;27000	0.0011
			Unlimited	0.00							
		No Makeup	250 ms	0.00							
			Unlimited	0.00							
Attractiveness	Intercept			3.13	0.17	18.93	40.81	<0.0001			
	ModelMakeup	Makeup		0.64	0.02	26.22	26506.05	<0.0001	1590.49	1;27000	<.0001
		No Makeup		0.00							
	length		250 ms	-0.04	0.11	-0.37	295.32	0.7115	0.07	1;270	0.7984
			Unlimited	0.00							
	ModelMakeup*length	Makeup	250 ms	0.03	0.03	0.80	26506.03	0.4240	0.64	1;27000	0.424
			Unlimited	0.00							
		No Makeup	250 ms	0.00							
			Unlimited	0.00							
Trustworthiness	Intercept			3.91	0.12	33.36	60.94	<0.0001			
	ModelMakeup	Makeup		0.00	0.02	0.13	26506.07	0.8988	12.13	1;27000	0.0005
		No Makeup		0.00							
	length		250 ms	-0.09	0.10	-0.88	302.96	0.3819	0.11	1;270	0.7389
			Unlimited	0.00							
	ModelMakeui*length	Makeup	250 ms	0.11	0.03	3.29	26506.04	0.0010	10.85	1;27000	0.001
			Unlimited	0.00							
		No Makeup	250 ms	0.00							
			Unlimited	0.00							

**Figure 2:**
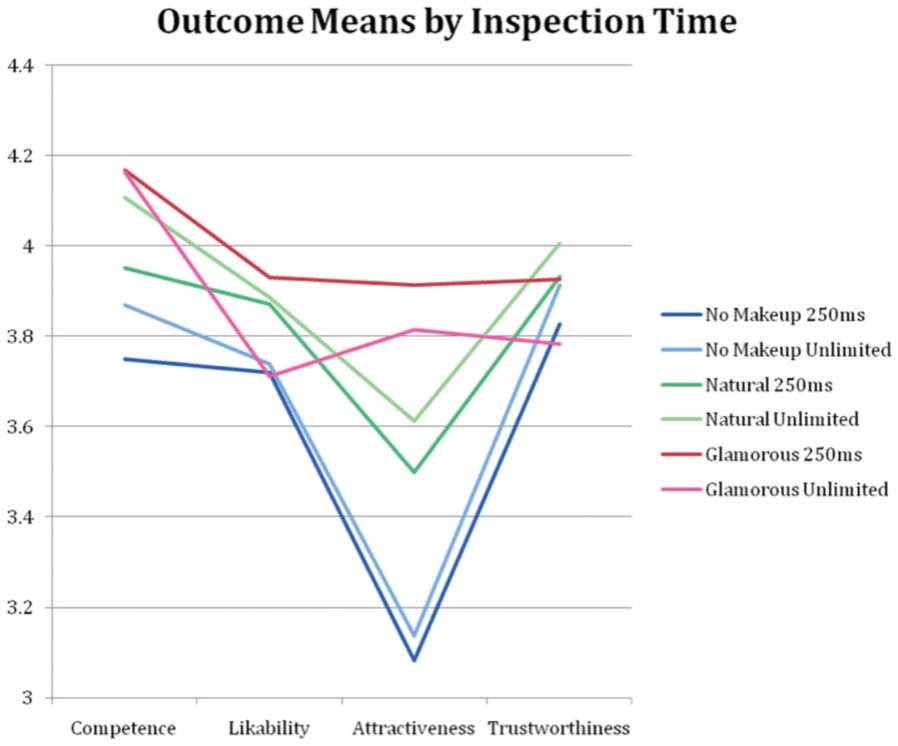
Means for the no makeup, natural and glamorous looks at 250 ms, and unlimited inspection times.

Analysis of the effects of the individual makeup “looks” revealed that each had a significant positive effect on judgments of competence and attractiveness both at 250 ms and at longer inspection times (Table 3). With unlimited inspection time, the natural and professional looks had significant positive effects on likability (natural t(26502) = 4.93, professional t(26502) = 5.18, both p<.0001, Bonferroni corrected significance level  = 0.05/24  = 0.002), while the glamorous look did not have a significant effect (t(26502) = -0.79, p = 0.4293). The natural look also had a significant positive effect on trustworthiness (t(26502) = 3.15, p = 0.0016), while the professional look did not have a significant effect (t(26502) = 1.50, p = 0.1337), and the glamorous look had a significant negative effect (t(26502) = -4.33, p<0.0001). There was a significant makeup x inspection time interaction for the glamorous look on all outcomes, with significantly larger positive effects when this look was presented for 250 ms than when presented with unlimited inspection time. The professional look showed significantly larger positive makeup effects at 250 ms for likeability (t(26502) = 2.4, p = 0.0162) and trustworthiness (t(26502) = 2.33, p = 0.0198), while the judgments of the natural look did not change significantly with inspection time (Table 4). Figure 2 illustrates these effects for 3 of the looks: the no makeup, natural and glamorous looks.

**Table 3:** Comparisons of mean outcome scores for the different makeup looks versus no makeup obtained from the regression models (see Table 4).

	Outcome	Contrast	Estimate	SE	t-Statistic	DF	P
**250 ms**	Competence	Glamorous vs No Makeup	0.42	0.03	16.03	26502	<0.0001
		Professional vs No Makeup	0.41	0.03	15.55	26502	<0.0001
		Natural vs No Makeup	0.2	0.03	7.64	26502	<0.0001
	Likability	Glamorous vs No Makeup	0.21	0.03	7.59	26502	<0.0001
		Professional vs	0.26	0.03	9.41	26502	<0.0001
		No Makeup					
		Natural vs No Makeup	0.15	0.03	5.45	26502	<0.0001
	Attractiveness	Glamorous vs No Makeup	0.83	0.03	31.19	26502	<0.0001
		Professional vs No Makeup	0.76	0.03	28.79	26502	<0.0001
		Natural vs No Makeup	0.4	0.03	15.28	26502	<0.0001
	Trustworthiness	Glamorous vs No Makeup	0.09	0.03	3.52	26502	0.0004
		Professional vs No Makeup	0.14	0.03	5.17	26502	<0.0001
		Natural vs No Makeup	0.1	0.03	3.76	26502	0.0002
**Unlimited**	Competence	Glamorous vs No Makeup	0.3	0.03	10.16	26502	<0.0001
		Professional vs No Makeup	0.37	0.03	12.54	26502	<0.0001
		Natural vs No Makeup	0.24	0.03	8.22	26502	<0.0001
	Likability	Glamorous vs No Makeup	-0.02	0.03	-0.79	26502	0.4293
		Professional vs No Makeup	0.16	0.03	5.18	26502	<0.0001
		Natural vs No Makeup	0.15	0.03	4.93	26502	<0.0001
	Attractiveness	Glamorous vs No Makeup	0.68	0.03	23.01	26502	<0.0001
		Professional vs No Makeup	0.76	0.03	25.56	26502	<0.0001
		Natural vs No Makeup	0.48	0.03	16.1	26502	<0.0001
	Trustworthiness	Glamorous vs No Makeup	-0.13	0.03	-4.33	26502	<0.0001
		Professional vs No Makeup	0.04	0.03	1.5	26502	0.1337
		Natural vs No Makeup	0.09	0.03	3.15	26502	0.0016

**Table 4:** Regression models for the makeup looks.

Outcome	Covariate			Estimate	SE	t-Stat.	DF	P	F-Stat.	Num DF	DenomDF	P
Competence	Intercept			3.87	0.12	33.53	65.93	<0.0001				
	Model Makeup	Glamorous		0.30	0.03	10.16	26502.03	<0.0001	162.05	3	27000	<0.0001
		Professional		0.37	0.03	12.54	26502.09	<0.0001				
		Natural		0.24	0.03	8.22	26502.03	<0.0001				
		No Makeup		0.00								
	Length		250 ms	-0.12	0.10	-1.24	299.97	0.2167	0.93	1	266	0.3369
			Unlimited	0.00								
	Model Makeup *Length	Glamorous	250 ms	0.12	0.04	3.11	26502.01	0.0019	6.26	3	27000	0.0003
			Unlimited	0.00								
		Professional	250 ms	0.04	0.04	1.01	26502.05	0.3119				
			Unlimited	0.00								
		Natural	250 ms	-0.04	0.04	-1.04	26502.02	0.2993				
			Unlimited	0.00								
		No Makeup	250 ms	0.00								
			Unlimited	0.00								
Likability	Intercept			3.74	0.14	26.50	44.18	<0.0001				
	Model Makeup	Glamorous		-0.02	0.03	-0.79	26502.02	0.4293	37.33	3	27000	<0.0001
		Professional		0.16	0.03	5.18	26502.09	<0.0001				
		Natural		0.15	0.03	4.93	26502.03	<0.0001				
		No Makeup		0.00								
	Length		250 ms	-0.01	0.10	-0.14	304.24	0.8893	0.52	1	266	0.4725
			Unlimited	0.00								
	Model Makeup *Length	Glamorous	250 ms	0.23	0.04	5.65	26502.01	<0.0001	14.43	3	27000	<0.0001
			Unlimited	0.00								
		Professional	250 ms	0.10	0.04	2.40	26502.05	0.0162				
			Unlimited	0.00								
		Natural	250 ms	0.00	0.04	-0.04	26502.02	0.9674				
			Unlimited	0.00								
		No Makeup	250 ms	0.00								
			Unlimited	0.00								
fAttractiveness	Intercept			3.13	0.17	18.93	40.80	<0.0001				
	Model Makeup	Glamorous		0.68	0.03	23.01	26502.02	<0.0001	650.34	3	27000	<0.0001
		Professional		0.76	0.03	25.56	26502.07	<0.0001				
		Natural		0.48	0.03	16.10	26502.02	<0.0001				
		No Makeup		0.00								
	Length		250 ms	-0.04	0.11	-0.37	294.88	0.7114	0.04	1	266	0.8450
			Unlimited	0.00								
	Model Makeup *Length	Glamorous	250 ms	0.14	0.04	3.65	26502.01	0.0003	10.42	3	27000	<0.0001
			Unlimited	0.00								
		Professional	250 ms	0.00	0.04	0.12	26502.04	0.9018				
			Unlimited	0.00								
		Natural	250 ms	-0.07	0.04	-1.81	26502.01	0.0701				
			Unlimited	0.00								
		No Makeup	250 ms	0.00								
			Unlimited	0.00								
Trustworthiness	Intercept			3.91	0.12	33.36	60.92	<0.0001				
	Model Makeup	Glamorous		-0.13	0.03	-4.33	26502.03	<0.0001	17.96	3	27000	<0.0001
		Professional		0.04	0.03	1.50	26502.10	0.1337				
		Natural		0.09	0.03	3.15	26502.03	0.0016				
		No Makeup		0.00								
	Length		250 ms	-0.09	0.10	-0.88	302.87	0.3819	0.00	1	266	0.9598
			Unlimited	0.00								
	Model Makeup *Length	Glamorous	250 ms	0.22	0.04	5.58	26502.01	<0.0001	13.51	3	27000	<0.0001
			Unlimited	0.00								
		Professional	250 ms	0.09	0.04	2.33	26502.06	0.0198				
			Unlimited	0.00								
		Natural	250 ms	0.01	0.04	0.16	26502.02	0.8699				
			Unlimited	0.00								
		No Makeup	250 ms	0.00								
			Unlimited	0.00								

Finally, to determine luminosity contrasts, color images were individually hand-labeled to define 4 regions: the eyes, including the eye itself (the sclera, iris, and pupil), eyelashes, the skin between the epicanthal fold and the eye; and the skin immediately below the eye; the lips; the annuli surrounding the eyes; and an annulus surrounding the lips. Then using a Spectra® PhoRad® Photometer, luminance values in candelas per meter squared (cd/m2) were collected from the iMac computer screen within the four regions and averaged, yielding mean luminance values for each of the four regions. The mean luminance for the eyes and lips were averaged to produce the mean feature luminance, and the mean luminance values for the eye and lip annuli were averaged to produce the mean skin luminance. Facial contrast was calculated as C_f_  =  (feature luminance – skin luminance)/(feature luminance + skin luminance) [26], [32].

Analyses confirmed that each pair of looks was significantly different in level of facial luminance contrast (all |t(72)|≥5.87, p<.0001, Bonferroni corrected significance level  = 0.05/18 = 0.003), except the natural look versus no makeup, which was marginally non-significant (t(72) = -1.83, p = 0.0717) (Tables S1 and S2). Facial luminance contrast increased from the face with no makeup to the face with natural, professional, and glamorous applications, respectively. Additional analyses not summarized here allowed for potential effect modification by race, but the interaction terms were not significant. All looks significantly decreased feature luminance (all |t(72)|≥4.19, p<0.0001), except the natural versus no makeup (t(72) = -.76, p = 0.4479), while none of the looks differed significantly from one another with respect to skin luminosity (all |t(72)|≤1.55, p>0.12).

In summary, we found evidence of significant effects of cosmetics in both studies. All the makeup looks significantly increased attractiveness and competence ratings at 250 ms and on longer inspection. Ratings of likeability and trust varied with makeup look and inspection time, suggesting that they elicited different reflexive and reflective responses.

## Discussion

Our results have a number of implications. As predicted, makeup had significant positive effects on ratings of female facial attractiveness at brief and longer inspection times. Ratings of competence increased significantly with makeup look tested on first glance and longer inspection. Effects were weaker and more variable for ratings of likability and trustworthiness, although generally positive.

Social psychologists have suggested that social warmth and social competence represent two universal dimensions of social perception by which we evaluate individuals and groups, [34] with warmth capturing traits related to social cooperation, and power/competence capturing cues relevant to advantage in social competition, such as status and dominance. Here we show a robust and positive effect of increased beauty on social power/competence and a generally positive but more nuanced and variable effect on social warmth.

Past studies have shown that attractive people are expected to do better on the job, in school, and in life – and are treated that way – by being agreed with, deferred to, helped, and granted larger personal space [7]. In a recent experimental study using a task for which physical attractiveness did not improve productivity, researchers demonstrated conclusively that employers expect physically attractive workers to perform better at their jobs and be more competent [35].

But, as sociologists Webster and Driskell noted when first proposing the idea of beauty as status, there are important differences between attractiveness and other status characteristics such as race or sex: beauty is a malleable characteristic [36]. They predicted that, given the powerful effect of status, “attractiveness will assume increasing significance as other characteristics such as race and sex fall into disuse.” We suggest that attractiveness has assumed increasing significance, and will continue to do so as long as beauty remains an often unconscious proxy for status and ability.

The beauty halo effect has been called the “what is beautiful is good” effect. In our study, makeup increased inferences of warmth and cooperation (likability and trustworthiness) when faces were presented very briefly, but did not always do so on longer inspection. In general, there is less agreement about whether beauty invariably signals social cooperation, with some studies suggesting that there is a ”dark side” to beauty characterized by vanity, immodesty, or greater likelihood to cheat on a partner [37]. Our findings suggest that it may be fruitful to disentangle the effects of beauty from beauty enhancement, or phenotype from extended phenotype here. It may be that natural beauty or natural appearing beauty leads to positive inferences of social cooperation, where more obvious beauty enhancement may lead to neutral or even negative inferences. Finally, our results provide additional evidence that judgments of facial trustworthiness and facial attractiveness are at least partially separable; the highest contrast makeup (glamorous) increased attractiveness significantly while at the same time decreasing judgments of trustworthiness.

Our study looked at one potential source of the cosmetics effect on face perception, increasing luminance contrast between the features (eyes and lips) and the surrounding skin, and looked for the first time at luminance contrast in African American and Hispanic faces. We found that cosmetics increased luminance contrast by significantly darkening the eyes and lips. Skin was neither significantly lightened nor darkened. However, luminance contrast effects for our natural look compared to a face without makeup was only marginally significant. It is likely that cosmetics induced image changes other than changes in luminance contrast contributed to our effects. These include possible changes in the smoothness of skin tone, in the redness of skin color or lip color, and in shading that accentuates the cheekbones. Previous research has shown that makeup can improve skin appearance, evenness, and texture to appear healthier, fertile, and youthful [38], [39], and that skin and lip color can contribute significantly to perception of sex typicality and attractiveness [40], [41], with lip redness enhancing femininity and attractiveness of female Caucasian faces [41].

Finally, our study included only North American subjects; we do not know if such effects will be found in subjects from other cultures.

In sum, we show that faces with cosmetics engage both fast, reflexive processes, and more deliberative conscious processes. The fast, automatic effects are uniformly strong and positive for all outcomes. In situations where a perceiver is under a high cognitive load or under time pressure, he or she is more likely to rely on such automatic judgments for decision-making. Facial images appear on ballots, job applications, websites and dating sites. Our results underscore the malleability of judgments derived from facial images of a single individual at zero acquaintance, judgments that can be highly consequential. When inferring trustworthiness, likeability, or competence from an image, we are influenced significantly not only by the attractiveness of the inherited phenotype but by the effects of the “extended phenotype,” in this case, makeup.

## Supporting Information

Table S1 Comparisons of mean luminosity measures for the different makeup looks obtained from the luminosity analysis regression models (see Table 2).
(DOCX)

Table S2 Regression models for the luminosity analysis.
(DOCX)
