# Supplementary material for: Cosmetics as a Feature of the Extended Human Phenotype: Modulation of the Perception of Biologically Important Facial Signals
Source: PLoS One. 2011 Oct 3;6(10):e25656. doi: 10.1371/journal.pone.0025656 (PMC3185017; doi:10.1371/journal.pone.0025656)
Supplement: Table S1 — Comparisons of mean luminosity measures for the different makeup looks obtained from the luminosity analysis regression models (see Table 2). (DOCX) [file pone.0025656.s001.docx]

**Table S1.** Comparisons of mean luminosity measures for the different makeup looks obtained from the luminosity analysis regression models (see Table 2)

| **Outcome** | **Look** | **Estimate** | **SE** | **t-Stat.** | **DF** | **P** |
| --- | --- | --- | --- | --- | --- | --- |
| Feature Luminosity | Glamorous vs. Professional | -9.90 | 2.36 | -4.19 | 72 | 0.0001 |
|  | Glamorous vs. Natural | -22.01 | 2.36 | -9.33 | 72 | <0.0001 |
|  | Glamorous vs. No Makeup | -23.81 | 2.36 | -10.09 | 72 | <0.0001 |
|  | Professional vs. Natural | -12.11 | 2.36 | -5.13 | 72 | <0.0001 |
|  | Professional vs. No Makeup | -13.91 | 2.36 | -5.89 | 72 | <0.0001 |
|  | Natural vs. No Makeup | -1.80 | 2.36 | -0.76 | 72 | 0.4479 |
| Skin Luminosity | Glamorous vs. Professional | -1.29 | 2.84 | -0.45 | 72 | 0.6505 |
|  | Glamorous vs. Natural | -4.41 | 2.84 | -1.55 | 72 | 0.1249 |
|  | Glamorous vs. No Makeup | -3.27 | 2.84 | -1.15 | 72 | 0.2543 |
|  | Professional vs. Natural | -3.12 | 2.84 | -1.10 | 72 | 0.2761 |
|  | Professional vs. No Makeup | -1.97 | 2.84 | -0.69 | 72 | 0.4898 |
|  | Natural vs. No Makeup | 1.15 | 2.84 | 0.40 | 72 | 0.6879 |
| Facial Luminance Contrast | Glamorous vs. Professional | -0.10 | 0.02 | -6.58 | 72 | <0.0001 |
|  | Glamorous vs. Natural | -0.19 | 0.02 | -12.45 | 72 | <0.0001 |
|  | Glamorous vs. No Makeup | -0.22 | 0.02 | -14.28 | 72 | <0.0001 |
|  | Professional vs. Natural | -0.09 | 0.02 | -5.87 | 72 | <0.0001 |
|  | Professional vs. No Makeup | -0.12 | 0.02 | -7.69 | 72 | <0.0001 |
|  | Natural vs. No Makeup | -0.03 | 0.02 | -1.83 | 72 | 0.0717 |
