# Supplementary material for: Cosmetics as a Feature of the Extended Human Phenotype: Modulation of the Perception of Biologically Important Facial Signals
Source: PLoS One. 2011 Oct 3;6(10):e25656. doi: 10.1371/journal.pone.0025656 (PMC3185017; doi:10.1371/journal.pone.0025656)
Supplement: Table S2 — Regression models for the luminosity analysis. (DOCX) [file pone.0025656.s002.docx]

**Table S2**. Regression models for the luminosity analysis

| **Outcome** | **Covariate** |  | **Estimate** | **SE** | **t-Stat.** | **DF** | **P** | **F-Stat.*** | **P** |
| --- | --- | --- | --- | --- | --- | --- | --- | --- | --- |
| Feature Luminosity | Intercept |  | 60.52 | 2.84 | 21.28 | 24 | <0.0001 |  |  |
|  | Look | Glamorous | -23.81 | 2.36 | -10.09 | 72 | <0.0001 | 44.67 | <0.0001 |
|  |  | Professional | -13.91 | 2.36 | -5.89 | 72 | <0.0001 |  |  |
|  |  | Natural | -1.80 | 2.36 | -0.76 | 72 | 0.4479 |  |  |
|  |  | No Makeup | Reference |  |  |  |  |  |  |
| Skin Luminosity | Intercept |  | 68.36 | 4.86 | 14.07 | 24 | <0.0001 |  |  |
|  | Look | Glamorous | -3.27 | 2.84 | -1.15 | 72 | 0.2543 | 0.96 | 0.4144 |
|  |  | Professional | -1.97 | 2.84 | -0.69 | 72 | 0.4898 |  |  |
|  |  | Natural | 1.15 | 2.84 | 0.40 | 72 | 0.6879 |  |  |
|  |  | No Makeup | Reference |  |  |  |  |  |  |
| Facial Contrast | Intercept |  | -0.04 | 0.02 | -1.69 | 24 | 0.1036 |  |  |
| Luminosity | Look | Glamorous | -0.22 | 0.02 | -14.28 | 72 | <0.0001 | 83.19 | <0.0001 |
|  |  | Professional | -0.12 | 0.02 | -7.69 | 72 | <0.0001 |  |  |
|  |  | Natural | -0.03 | 0.02 | -1.83 | 72 | 0.0717 |  |  |
|  |  | No Makeup | Reference |  |  |  |  |  |  |

*Numerator DF = 3, denominator DF = 72
